# Artificial selection for host resistance to tumour growth and subsequent cancer cell adaptations: an evolutionary arms race

**DOI:** 10.1038/s41416-020-01110-1

**Published:** 2020-10-07

**Authors:** Arig Ibrahim-Hashim, Kimberly Luddy, Dominique Abrahams, Pedro Enriquez-Navas, Sultan Damgaci, Jiqiang Yao, Tingan Chen, Marilyn M. Bui, Robert J. Gillies, Cliona O’Farrelly, Christina L. Richards, Joel S. Brown, Robert A. Gatenby

**Affiliations:** 1 Cancer Biology and Evolution Program, H. Lee Moffitt Cancer Center, Tampa, FL USA; 2 Department of Cancer Physiology, H. Lee Moffitt Cancer Center, Tampa, FL USA; 3 Department of Integrative Biology, University of South Florida, Tampa, FL USA; 4 School of Biochemistry and Immunology, Trinity College Dublin, Dublin, Ireland; 5 Department of Biostatistics & Bioinformatics, H. Lee Moffitt Cancer Center, Tampa, FL USA; 6 Analytic Microscopy Core, H. Lee Moffitt Cancer Center, Tampa, FL USA; 7 Department of Pathology, H. Lee Moffitt Cancer Center, Tampa, FL USA; 8 Department of Radiology, H. Lee Moffitt Cancer Center, Tampa, FL USA; 9 Department of Integrated Mathematical Oncology, H. Lee Moffitt Cancer Center, Tampa, FL USA; 10 Department of Biological Sciences, University of Illinois, at Chicago, Chicago, IL USA

**Keywords:** Cancer genomics, Experimental evolution

## Abstract

**Background:**

Cancer progression is governed by evolutionary dynamics in both the tumour population and its host. Since cancers die with the host, each new population of cancer cells must reinvent strategies to overcome the host’s heritable defences. In contrast, host species evolve defence strategies over generations if tumour development limits procreation.

**Methods:**

We investigate this “evolutionary arms race” through intentional breeding of immunodeficient SCID and immunocompetent Black/6 mice to evolve increased tumour suppression. Over 10 generations, we injected Lewis lung mouse carcinoma cells [LL/2-Luc-M38] and selectively bred the two individuals with the slowest tumour growth at day 11. Their male progeny were hosts in the subsequent round.

**Results:**

The evolved SCID mice suppressed tumour growth through biomechanical restriction from increased mesenchymal proliferation, and the evolved Black/6 mice suppressed tumour growth by increasing immune-mediated killing of cancer cells. However, transcriptomic changes of multicellular tissue organisation and function genes allowed LL/2-Luc-M38 cells to adapt through increased matrix remodelling in SCID mice, and reduced angiogenesis, increased energy utilisation and accelerated proliferation in Black/6 mice.

**Conclusion:**

Host species can rapidly evolve both immunologic and non-immunologic tumour defences. However, cancer cell plasticity allows effective phenotypic and population-based counter strategies.

## Background

Cancers typically consist of heterogeneous competing cellular subpopulations that interact with each other and their environment^1^ so that cell phenotypes optimally adapted to local environmental conditions will proliferate at the expense of those less fit. Since tumour populations die with the host, each new cancer must reinvent strategies to overcome the host’s defences. In contrast, host species can evolve strategies to suppress cancer growth over generations if tumour development limits procreation. These competing dynamics are described as an “evolutionary arms race.”

The description of cancer as an evolutionary system, first proposed by Cairns and Nowell more than 60 years ago,^2,3^ has been well-recognised particularly in the application of evolutionary biology to understand cancer progression and resistance to therapy.^1,4–6^ In diverse species of hosts, cancer suppressor mechanisms have been examined, particularly in the context of cancer incidence and body size (e.g. Peto’s Paradox^7^). However, we are not aware of prior studies that explicitly applied selection to increase an animal species’ resistance to cancer.

Evolution can be driven by human or “artificial” selection through intentional breeding of domesticated animals for desirable traits that are evolutionarily feasible within the underlying genome.^8^ For example, the phenotypic and genetic diversity of the domestic dog diversified from that of the wolf genome. Human intervention can also exert unintentional selection on organisms, leading to dramatic evolutionary changes such as antibiotic or pesticide resistance.^9^

Here we hypothesise that, with the application of appropriate selection forces, laboratory animals can evolve phenotypes that are resistant to the growth of implanted tumours, providing insights into available tumour-suppression strategies. To examine the range of potential tumour-suppression strategies, we examined the artificial evolution of resistance in immunocompetent and immunodeficient mice.

## Methods

### Animal models and care

All animal studies completed were approved and maintained under University of South Florida’s Institutional Animal Care and Use Committee (IACUC) at H. Lee Moffitt Cancer Center (protocol reference numbers 2014R, 0061R and 3735R). Animals were maintained in accordance with IACUC standards of care in pathogen-free rooms, in the USF Vivarium on site at the Moffitt Cancer Research Center (Tampa, FL). For bioluminescence imaging, isoflurane was used as anaesthesia for the mice. For effect, the mice were induced under 3% isoflurane and maintained for the duration of the imaging at 1.5% isoflurane. Oxygen flow rates were kept between 0.25 and 0.5 L/min.

CO_2_ inhalant is used for humane euthanasia. During euthanasia, mice were placed in a chamber or kept in their home cage when possible. CO_2_ was turned on with the use of a flow meter that ensured an appropriate displacement rate of air inside the enclosure by displacing 10–30% of the chamber volume per minute. A secondary physical method of euthanasia was completed by cervical dislocation.

We used both C57BL/6 and SCID/beige mice accessions. The original breeding pairs for both accessions were acquired from Charles River (Wilmington, MA). From each generation, 10 weaned C57BL/6 (Black/6) and 10 weaned SCID/beige (SCID) male mice were injected on the right flank with LL/2-luc-M38 cells at 6 weeks of age. Females from the same pool of offspring as the males were kept for breeding.

Because the LL/2-luc-M38 cells grow very rapidly, often killing the host by day 25, we selected animals for breeding based on the tumour growth at day 11 following injection. This was necessary to allow sufficient time for the animal to breed successfully (i.e. prior to the time in which the tumour burden precluded successful mating). The male with the smallest tumour of the 10 injected mice was bred with 4 females, and the male with the next smallest tumour was bred with two other females. This procedure was repeated for 10 generations. Mice were imaged with the In Vivo Imaging System 200 (IVIS-Spectrum 200, Caliper Life Sciences, Waltham, Massachusetts) at the time of injection (time 0) and on days 7 and 14 post injections. Tumours were also measured with callipers twice a week. In some experiments after generation 10, we continued to measure tumours until day 28 to examine the durability of the host tumour suppression.

To explore the mechanisms underlining the generated evolved mice, we purchased 10 males of C57BL/6 and 10 males of SCID mice. These are referred to as Non-evolved Black/6 and Non-evolved SCID. At 6 weeks of age, we injected 10 mice of each accession subcutaneously into the right flank with LL/2-luc-M38 cells. At the same time, we injected LL/2-luc-M38 cells into 10 male mice from generation 14 of the selection experiment. These are referred to as Evolved Black/6 and Evolved SCID mice. Tumour volumes were measured with a calliper twice weekly up to 18 days. Tumours were collected from five of the animals at 11 days, and the remaining tumours were collected at 28 days.

### Cell culture and inoculation

LL/2-luc-M38 is a bioluminescent cell line of Lewis lung carcinoma cells, which were derived from a spontaneous lung tumour in C57BL/6 mice. We obtained these cell lines from Xenogen Corporation (Alameda, CA). Cells were authenticated by short tandem-repeat analysis and confirmed to be free of mycoplasma. The LL/2-luc-M38 cells were grown in DMEM/F12 supplemented with 10% foetal bovine serum (HyClone) and 1% pen strep (Corning) in 5% CO_2_. To be certain the observed effects were due to host evolution and not tumour cell evolution, we expanded the original LL/2-luc-M38 cells through in vitro cell culture prior to the experiments, divided this population into aliquots of 5 × 10^5^ cells in 200 µL of PBS and stored them at −80 °C. For injections, the hair was removed from the right flank of each mouse, and the 200-µl cell suspension from these aliquots was slowly injected with a 27-G needle.

For the experimental metastasis model, 200 µL containing 5 × 10^5^ cells in PBS were injected directly and slowly (over the course of 1 min) into the tail vein of each mouse, and cell distributions were verified by bioluminescent imaging immediately following injection.

### Bioluminescent imaging

In vivo bioluminescence imaging was completed with the Xenogen IVIS-200 System (Caliper Life Sciences, Hopkinton, MA) as previously published.^10^ Prior to each IVIS imaging session, mice were intraperitoneally injected with sterile d-luciferin at 10 µl per gram body weight. D-luciferin was prepared in PBS at 15 mg/ml. After tumour injection, the mice were placed inside of an oxygen- rich induction chamber consisting of 2.5% isoflurane (Henry Schein, Melville, NY). The mice were then imaged 5 min post injection. Mice were placed on their left side on the imaging platform. Anaesthesia was maintained using nose cones with a 1.5–2% isoflurane flow rate. The IVIS imaging chamber consists of a warming platform and a cryogenically cooled CCD camera to capture both a visible-light photograph of the animals and a bioluminescent image. Data were acquired and analysed utilising the Living Imaging 4.3.1 software. Regions of interest were placed around each tumour to assess the photon intensity, in units of photons/second (p/s).

### Tumour cell isolation

Tumours were collected from mice post-mortem and placed in cold DMEM with 5% penicillin–streptomycin. Tumours were processed immediately after resection by mechanical disaggregation. Tumour tissues were placed in sterile 10-cm culture dishes, washed in DPBS, and excess tissue, including adipose and skin, was removed. Tumours were minced into 2–3-mm fragments and placed in a sterile tissue sieve with 44-mm nylon mesh. DMEM with 10% FBS was added, and the tissues were disaggregated by mechanical pressure using the sterile blunt end of a syringe handle. The resulting single-cell suspension was cultured at 37 °C with 5% CO_2_. Media was changed daily, and cells were frozen after 2–3 passages in FBS with 10% DMSO. For microarray analyses, cells were later thawed and passaged 3 times prior to RNA isolation for microarray analyses.

### Splenocyte survival and tumour cell-killing assay

Spleens were removed from mice post-mortem and placed in 5 ml of RPMI-1640 containing 10% penn/strep in 15-ml conical tubes and transported on ice. Spleens were then placed in 10-cm Petri dishes and washed with phosphate buffer saline (PBS). Mechanical disaggregation was performed using 100-micron mesh and a syringe piston. Tissues were washed with PBS and filtered through a 70-micron filter to achieve a single-cell suspension and centrifuged at 1500 RPM for 5 min. To remove red blood cells, pellets were resuspended in 15 ml of ACK (ammonium–chloride–potassium) lysing buffer (ThermoFisher Scientific) for 15 min with intermittent agitation. Thirty millilitres of RPMI-1640 containing 10% foetal bovine serum was added, and cells were spun down to remove lysis buffer. Following two washes with 10 ml of RPMI-1640, splenocytes were counted and rested for 2 h in RPMI-1640 containing 10% foetal bovine serum at 37 °C with 5% CO_2_.

LL/2-luc-M38 cells were thawed and passaged twice. After removal from the flask with 0.5% Trypsin-EDTA, cells were washed in RPMI-1640 with 10% FBS and counted. Cells were fluorescently labelled with 1 µM CellTrace Violet (ThermoFisher) in PBS at a concentration of 1 × 10^6^ cells per ml for 20 min at 37 °C. RPMI-1640 with 10% FBS was added for 5 min to remove any free dye. Cells were then washed and counted.

Unstained splenocytes and fluorescently labelled LL/2-luc-M38 cells were co-cultured at a 20:1 effector-to-target-cell ratio for 24 h. Suspended and adherent cells were collected and counted. Additionally, cells were stained with propidium iodide (PI) and analysed by flow cytometry (BD LSR II, BD Bioscience). Splenocyte cell number was determined by multiplying the frequency of PI- negative, CellTrace Violet negative cells by the total live cells counted. Tumour cell number was calculated by multiplying the frequency of PI-negative, CellTrace Violet positive cells by the total live cells counted. Percent killing was calculated as [(untreated − treated)/untreated] × 100.

### Complete blood counts (CBC)

CBC was performed following standard protocols. Blood was collected post-mortem from non-tumour-bearing mice via cardiac puncture. This terminal collection was completed by laying the animal on its back and inserting the syringe vertically through the sternum. Approximately 300–350 µl was collected and placed into an EDTA tube. Analysis was performed using a Heska HemaTrue^TM^ analyser.

### Immune panel on peripheral blood

Blood was collected post-mortem via cardiac puncture. Red blood cells were lysed in 15 ml of ACK lysing buffer (ThermoFisher Scientific) for 15 min with intermittent agitation. About 30 ml of RPMI-1640 containing 10% foetal bovine serum was added, and cells were spun down to remove lysis buffer. The remaining cells were stained in PBS containing 0.5% BSA and 0.1% sodium azide with Ly-6G/C FITC, NK1.1 APC, CD3e APC-Cy7 and CD19 V450 for 1 h at 4 °C. Samples were analysed using BD LSR II flow cytometer with Diva acquisition software. FlowJo (Treestar) analysis software was used with standard gating practices to remove debris and doublets (Supplementary Fig. S1a–c).

### Immunohistochemistry

Tissue slides were stained using a Ventana Discovery XT automated system (Ventana Medical Systems, Tucson, AZ) as per the manufacturer’s protocol with proprietary reagents. Slides were deparaffinised on the automated system with EZ Cell Conditioning 1 (Ventana). The detection system used was the Ventana OmniMap kit. Slides were then counterstained with haematoxylin. Next, the slides were dehydrated and coverslipped as per the normal laboratory protocol. The rabbit primary antibody that reacts with mouse CD31 (#ab28364, Abcam, Cambridge, MA) was used at a 1:200 concentration in Dako antibody diluent (Carpenteria, CA) and incubated for 32 min. The Ventana Anti-Rabbit Secondary Antibody was used for 20 min. The rabbit primary antibody that reacts with mouse Cleaved Caspase 3 (#9661, Cell Signaling, Danvers, MA) was used at 1:2000 concentration in Dako antibody diluent and incubated for 60 min. The Ventana Anti-Rabbit Secondary Antibody was used for 16 min. The rabbit primary antibody that reacts with mouse Ki67 (M3060, Spring Biosciences, and Pleasanton, CA) was used at a 1:100 concentration in Dako antibody diluent and incubated for 32 min. The Ventana OmniMap Anti-Rabbit Secondary Antibody was used for 20 min.

### Image analysis

An Aperio (Vista, CA) Positive Pixel Count® v9.0 algorithm software with the following thresholds: [Hue value = 0.1; Hue width = 0.5; colour saturation threshold = 0.04; IWP(High) = 220; Iwp(Low) = Ip(High) = 175; Ip(Low) = Isp(High) = 100 Isp(Low) = 0] was used to segment positive staining of various intensities. The algorithm was applied to the entire digital core image to determine the percentage of positive biomarker staining by the applicable area. The percentage of positive pixels (sum of weakly positive, positive and strongly positive divided by total pixels) in the applicable viable tumour area (designated by excluding necrotic volumes identified on H&E images) was then calculated.

### Second harmonic generation imaging

Second harmonic generation (SHG) images were captured through a ×25/0.95NA water objective lens with a Leica SP5 Multiphoton Microscope (Leica Microsystem GmbH, Wetzlar, Germany) equipped with a MaiTai DeepSee Ti-sapphire laser (Spectra-Physics Inc., Mountain View, CA) and HyD detectors. The MP laser was tuned to 880 nm, and emissions were collected through a 440-nm band-pass filter to achieve SHG imaging. In addition to SHG, bright-field images of the identical fields were captured using an Argon laser tuned to 488 nm and transmitted light PMT. All images and overlays were prepared in Leica LASAF software (Leica Microsystems GmbH, Wetzlar, Germany). Using Definiens Developer version 2.4 (Definiens AG, Munich, Germany), SHG signals from each image were evaluated. ROI was drawn at the edge and the core of the tissue along the observed interface of differential tissue morphology. An autothreshold algorithm was used to segment the SHG signal from the background within the edge and the core regions. Then, the total fluorescence signal from SHG was determined for each region in each image. Finally, SHG signal per tissue area was calculated by using the bright-field image to determine the total area of the region.

### Isolation of RNA and sample processing for microarray analysis

Total RNA from mouse tumours and cells was isolated and purified using the RNeasy clean-up procedure (Qiagen Inc., Valencia, CA). The quality of total RNA was assessed by agarose gel electrophoresis and A260/A280 ratio using the NanoDrop spectrophotometer (Thermo Scientific, Wilmington, DE, USA). One-hundred nanograms of total RNA served as the mRNA source for microarray analysis. The poly (A) RNA was converted to cDNA and then amplified and labelled with biotin using the Ambion Message Amp Premier RNA Amplification Kit (Life Technologies, Grand Island, NY) following the manufacturer’s protocol initially described by Van Gelder et al.^11^ Hybridisation with the biotin-labelled RNA, staining and scanning of the chips followed the procedure prescribed in the Affymetrix technical manual (Affymetrix, Santa Clara, California, USA). Scanned output files were processed using Affymetrix Expression Console software. The RNA isolation and microarray processing were performed by the Molecular Genomics Core at the Moffitt Cancer Center.

### Probe arrays

We used the GeneChip® Mouse Genome 430 2.0 Array to measure gene expression in the LL/2-Luc-M38 tumours. This array contains over 45,000 probesets designed from GenBank, dbEST and RefSeq sequences that were clustered based on build 107 of the UniGene database. The clusters were further refined by comparison to the publicly available draft assembly of the mouse genome. An estimated 39,000 distinct transcripts were detected, including over 34,000 well-substantiated mouse genes. Each gene is represented by a series of oligonucleotide probes that are identical to the sequence in the gene (PM probe) as well as oligonucleotides that contain a homomeric (base transversion) mismatch at the central base position of the oligomer (MM probe); this measures cross-hybridisation.

### Microarray analysis

Quality control of arrays was made by generating the following plots: PCA, NUSE, RLE, Density, Intensity and RNA degradation, and analysis was done with in-house R(3.1.1) scripts. Data were normalised using Robust Multi-array Average (RMA) algorithm. One outlier sample was defined in PCA plots as a sample that did not cluster together with the rest of the replicate samples. Instead, the outlier sample was clustered into another group. Only samples that passed all filters were selected for further analysis. Principal component analysis (PCA) was performed using Evince V2.7.0 (UmBio AB, Umeå, Sweden).

On the normalised and filtered data, we used principal variance component analyses [PVCA, 13–15] in JMP/Genomics (Version 8 for Windows, SAS Institute, Cary, NC, USA) to examine global expression trends in the LL/2-Luc-M38 tumours that were associated with host accession and level of host selection. The PVCA approach first reduces the dimensionality of the data set with PCA, and then computes variance components by fitting a mixed linear model to each principal component, treating each factor of interest in the model as a random effect (including continuous variables). We used the model PCi = accession + selection level + accession-by-selection level + error, where i indicates each principal component, starting with 1 and continuing through all principal components calculated in the PCA. The variance component for each factor is obtained by a weighted averaging across the values calculated for each principal component, weighted by the eigenvalues for the corresponding principal component. We used the same factors in a mixed model ANOVA to directly fit the model to gene expression (ANOVA model: gene expression = accession + selection level + accession-by-selection level + error).

To identify the probesets that can differentiate Non-evolved SCID from Evolved SCID at 11 days, we initially made PCA plots with all probesets. Then, select those probesets whose absolute loading values are greater than 0.02. In the final PCA plots, the first component captured 89.9% of variance and the second 7.18%. We made heatmaps based on the expression values of the selected probesets. To get log2-fold change between Non-evolved SCID and Evolved SCID, we used the average values of Non-evolved SCID and Evolved SCID groups: log2fc = average (log2NESD) – average (log2ESD). The probesets were split into up- (>0.5850) and downregulated (<0.5850) groups according to the log2fc value (i.e. a minimum 1.5× fold change). Probesets were annotated with Mouse430_2.na36.annot.csv (create date 03–30–2016). We further analysed up- and downregulated genes with MetaCore™ (Bioinformatics software from Thomson Reuters, https://portal.genego.com/) for significant pathway analysis and GO TERM enrichment analysis.

### Quantitative real-time PCR

All qRT-PCRs were performed on a 7900HT Fast-Real-Time System (Life Technologies Applied Biosystems®) using an iScript Real-Rime PCR kit with SYBR Green (BioRad, 170-8893). GAPDH expression was used as an internal control. Using RNeasy Plus Mini Kit (Qiagen), RNA was isolated from cancer cells isolated from the primary tumours of non-evolved SCID—11 days and evolved SCID—11 days. About 100 ng of mRNA was used per 20-µL reaction. The mRNA expression levels were normalised by calculating the ratios against GAPDH expression levels. Primers for Collagen 12a1 (Col12a1) F-5′-ACCCACCTTCCGACTTGAATT-3′, R- 5′-TAGGCCCATCTGTTGTAGGG-3′ were obtained from integrated DNA Technologies (Coralville, IA)38.

### Quantification of lung metastasis volume with MRI

Ex vivo images of lungs from Non-evolved and Evolved mice were obtained using T2-weighted pulse sequence in a 7 Tesla Biospec (Bruker Biospin Inc.). A 35-mm Litzcage coil (Doty Scientific) was used to carry out axial T2-weighted fast spin-echo multi-slice experiments (acquired with TE/TR [echo time/repetition time] = 31 ms/1700 ms, field of view (FOV) = 20 × 20 mm^2^, matrix = 256 × 256, yielding a spatial in-plane resolution of 78 µm and slice thickness of 0.5 mm). Tissue slices covered all the tissue, and there were no gaps between sequential slices. Images were quantified by drawing ROIs manually and using an implemented home-made code in AEDES software (Aedes, http://aedes.uef.fi/).

### Statistical analysis of tumour growth

We used ANCOVA (SYSTAT v13) to compare the effects of mouse accession and generation on tumour growth. In separate analyses, we used tumour size at week 7 (logarithmically transformed) and at week 14 (also log transformed) as the dependent variable. As independent variables, mouse accession was a categorical variable and mouse generation was a covariate. If slopes were homogeneous across groups (non-significant interaction of mouse accession and generation at *p* > 0.1), then the analysis was rerun without the interaction effect. This was the case for tumour size at week 7, but not so for tumour size at week 14. So, we ran separate least-squares regressions (SYSTAT v13) of generations of selection on week-14 log tumour sizes for the SCID and Black/6 mice. For additional tests of means, we used Student’s *t* tests where a *p* value of <0.05 was considered statistically significant (Prism 5 software).

## Results

### The mice evolved

**Fig. 1:**
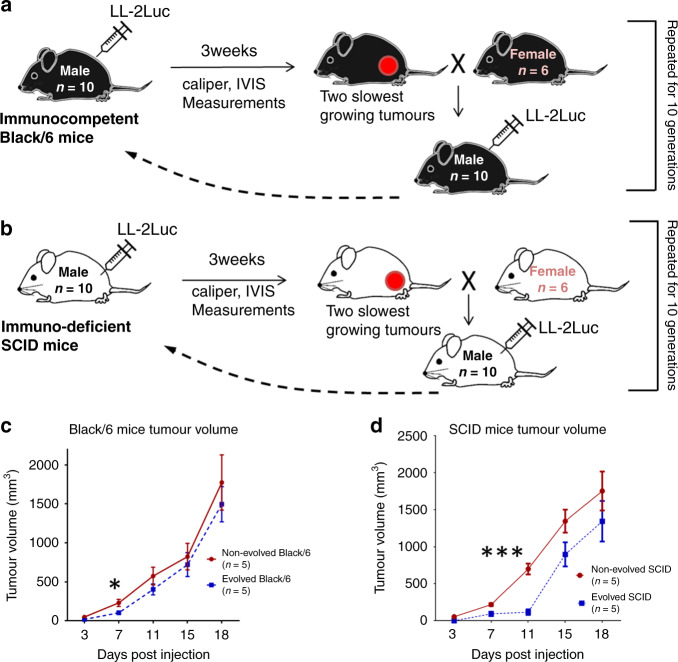
Experimental design for selection of Evolved mice, and tumour growth. **a** Evolved Black/6 (*n* = 10, male). **b** Evolved SCID mice (*n* = 10, male). **c** Comparison of tumour growth in non-evolved Black/6 mice (*n* = 5) and in evolved Black/6 mice (*n* = 5). **d** Comparison of tumour growth in Non-evolved SCID mice (*n* = 5) and in Evolved SCID mice (*n* = 5). There was a significant difference in the tumour growth starting at 3 days post injection of non-evolved and evolved Black/6 mice (*p = 0.01) but not after 7 days. There was a significant difference in tumour growth at 11 days post injection of Non-evolved and evolved SCID mice (***p = 0.001), but not at 15 days. Mean ± SEM is plotted with significance based on two-tailed unpaired Student’s *t* test.

Here we applied artificial selection to immunocompetent (C57BL/6, also named Black/6) and immune-deficient (SCID) mice to investigate evolutionarily available cancer-suppression strategies. We injected Lewis lung carcinoma cells (LL/2-Luc-M38) freshly prepared from frozen stocks subcutaneously into 6-week-old male mice. The two recipient mice with the slowest tumour growth were then bred with females from the same generation (Fig. 1a, b), and male mice from these litters were subsequently inoculated with LL/2 cells. Each cycle required 2.5–3.0 months to complete. The final tumour sizes were larger in the SCID mice than in immunocompetent Black/6 mice (*F*_1,195_ = 9.62, *p* < 0.002). We observed rapid evolution of increased resistance to tumour growth in both mouse accessions. Artificial selection over 10 generations resulted in a significant reduction of tumour size at day 7 (*F*_1,195_ = 17.16, *p* < 0.001). However, tumour growth in Evolved Black/6 mice accelerated after day 7 so that the tumour size at day 11 was not significantly different from control (slope of −0.092, *t*_90_ = 0.44, adjusted *r*^2^ = 0.013) (Supplementary Fig. S2a, b). In contrast, tumour suppression persisted in the SCID mice through day 11, and this growth inhibition increased with each generation (slope of −0.847, *t*_104_ = 5.5, adjusted *r*^2^ = 0.255). To examine the durability of the host strategy for tumour suppression, we measured the tumour beyond the usual selection date (i.e. day 11). During this period, tumour growth in the Evolved mice accelerated and was not different in size from the control group (i.e. non-evolved mice) (Fig. 1c, d).

### Altered host responses in the evolved mice

Under artificial selection pressure, both mouse accessions evolved enhanced mechanisms of tumour suppression. Evolved immunocompetent Black/6 mice demonstrated increased immune responses to the LL/2-Luc-M38 cells, while the evolved immune-deficient SCID mice increased biomechanical suppression of tumour growth through fibrous encapsulation.

**Fig. 2:**
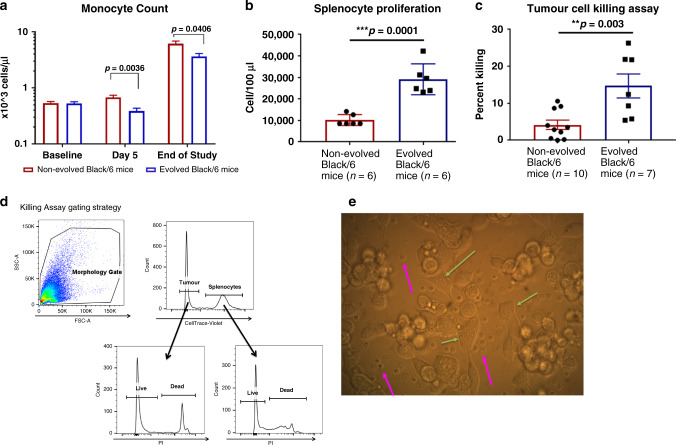
Strategy of evolved Black/6 mice. **a** Monocyte count in non-evolved and evolved Black/6 demonstrating a significant decrease in evolved mice at day 5 and at the end of the study (*p* < 0.04). **b**, **c** Ex vivo tumour cell-killing assays demonstrated a significant increase in the number of surviving splenocytes (*p* = 0.0001), and higher percent of tumour cell death in evolved Black/6 mice compared to non-evolved (*p* = 0.003). **d** Gating strategy for splenocyte survival and functional assays. **e** Representative image of the functional assay; fluorescently labelled splenocytes cultured with tumour cells are shown in pink arrows, and tumour cells shown in green arrows.

Black/6 mice have intact innate and adaptive immune systems. Therefore, Evolved Black/6 mice were examined for changes in immune-cell frequency, phenotype and function. Complete blood counts, flow cytometric immune phenotyping and ex vivo functional assays were performed comparing non-evolved and evolved Black/6 mice. Prior to tumour injection, evolved and non-evolved Black/6 mice did not differ in monocyte counts. However, 5 days post injection and at the end of the study (28 days), circulating monocytes were significantly lower in the evolved than non-evolved Black/6 mice (*p* = 0.0036 and *p* = 0.04, respectively) (Fig. 2a). Survival and killing capacity of splenocytes isolated from tumour-resistant evolved Black/6 mice were significantly higher than non-evolved mice (*p* = 0.0001, and *p* = 0.003, respectively) (Fig. 2b–e). No significant changes in circulating white blood cell, lymphocyte or granulocyte counts could be detected during tumour growth (Supplementary Fig. S3a–c). There were no changes in NK1.1^+^, CD19^+^ or Gr1^+^ cells detectable by flow cytometry. However, circulating CD3e^+^ T cells were significantly lower (*p* = 0.0172) in the evolved Black/6 mice than in the non-evolved mice at the end of the study (Supplementary Fig. S3d–g).

**Fig. 3:**
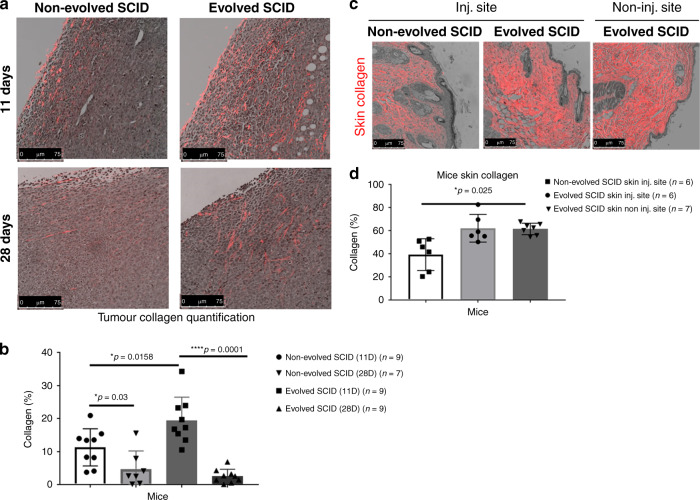
Strategy of evolved SCID mice. **a** Representative images of tumour collagen fibrillary structure in non-evolved and evolved SCID at 11 days and at 28 days post tumour inoculation. On the top of each image is a schematic presentation of the model showing the acclimation of the tumour cells to the increased collagen at the early time point of Evolved SCID mice. **b** Quantitative analysis of the tumour collagen in non-evolved and evolved SCID at 11 days and 28 days, showing significant differences at 11 days between non-evolved and evolved (*p* = 0.0158), between 11 and 28 days of non-evolved (*p* = 0.03) and between 11 and 28 days of evolved (*p* = 0.0001). **c** Representative images of skin collagen fibrillary structure in non-evolved and evolved SCID at the injection site and evolved non-injection site. **d** Quantitative analysis of the skin collagen showing a significant increase (*p = 0.025) between evolved and non-evolved mice on the injected sites. Mean ± SEM is plotted with significance based on two-tailed unpaired *t* test.

In contrast to Black/6 mice, severe combined immunodeficiency (SCID) mice cannot mount an adaptive immune response, thus limiting their options for restricting tumour growth to innate macrophage and neutrophil-mediated immunity (Supplementary Fig. S4a, b). The killing capacity of splenocytes isolated from non-evolved and evolved SCID mice did not differ significantly (Supplementary Fig. S5a, b). Immune-deficient SCID mice were observed to suppress tumour growth via mechanical restriction with increased collagen deposition in and around the tumour. We found collagen content in the stroma surrounding 11-day-old tumours to be significantly higher (*p* = 0.0158) in the evolved tumour-resistant SCID mice than in the non-evolved mice. To examine the durability of the host strategy for tumour suppression, collagen deposition was quantified beyond 11 days, and no increase in collagen deposition in evolved tumour-resistance mice was observed (Fig. 3a, b). Collagen was significantly increased at the tumour injection site as well as in the opposite (uninjected) flank (*p* = 0.025) in the non-tumour-bearing evolved SCID mice (Fig. 3c, d). Encapsulation or walling off injured and infected tissues is a host defence strategy to isolate and suppress pathogen growth in organisms lacking adaptive immune responses, including lower invertebrates and many plant species.^12,13^ Furthermore, tumour encapsulation^14,15^ and fibroblast infiltration^16^ (desmoplasia) is frequently observed clinically.

### Tumour cell counter strategies

Having shown that artificial selection promotes the evolution of host tumour suppression during the experimental period, we then investigated the durability of this response. Does the evolved host tumour suppression persist, or do the cancer cells evolve counter strategies? We therefore investigated the phenotypes of LL/2 tumour cells that were grown as tumours in Evolved or Non-evolved Black/6 or SCID mice using immunohistochemistry and microarray analysis.

**Fig. 4:**
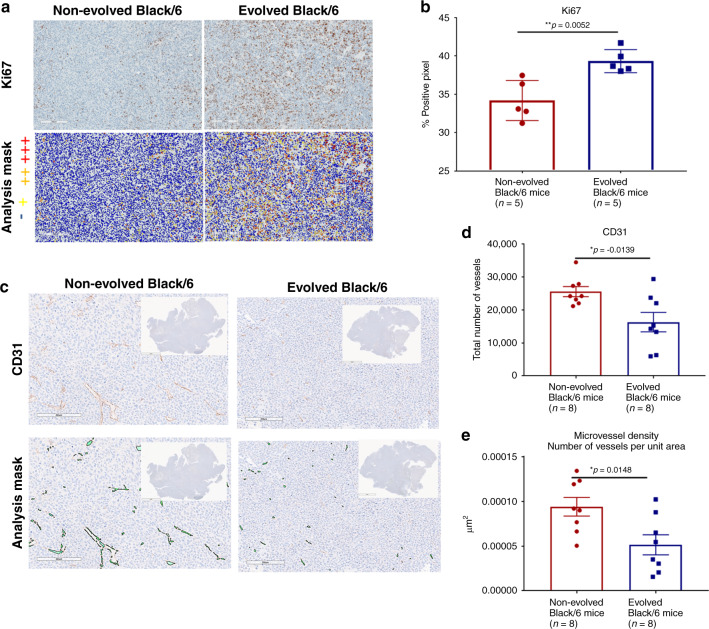
Counterstrategy of tumour cells at 11 days post injection into Black/6 mice. **a** Immunohistochemistry staining and **b** quantification of Ki67 (*n* = 5). Representative images of the tumour (upper panel) as well as a positive mask (lower panel). Percent Ki67-positive pixels were quantified over the entire viable area of tumour cross-section. Mean ± SEM is plotted**. c** Representative images of immunohistochemical CD31 staining of evolved and non-evolved Black/6 tumour tissue. Scale bar in IHC images is 200 µm. Quantification of **d** the total number of vessels and **e** microvessel density (MVD) across the whole image, demonstrating significant decrease in the total number of vessels (*p = 0.0139) and the number of vessels per unit area (*p = 0.0148) in evolved compared to non-evolved Black/6 mice. Mean ± SEM is plotted with significance based on two-tailed unpaired Student’s *t* test.

The Evolved Black/6 mice demonstrated peak tumour suppression (tumour growth compared to that of Non-evolved mice) at day 7. By day 11, the tumour size of LL/2-Luc-M38 cancer cells in the Evolved mice rapidly approached the tumour size in the non-evolved mice. The LL/2-Luc-M38 cells growing in the evolved Black/6 mice displayed two adaptive strategies—faster proliferation (Fig. 4a, b), and decreased angiogenesis (Fig. 4c–e) compared to the parental cell line growing in non-evolved Black/6 mice.

On the other hand, in the Evolved SCID mice, LL/2-Luc-M38 cells displayed lower cell proliferation and higher necrosis as a response to mechanical pressures by collagen (Supplementary Fig. S6a–c). No difference in angiogenesis was observed (Supplementary Fig. S7a–c). In addition, the LL/2-Luc-M38 rapidly altered their phenotype to increase degradation of the increased extracellular matrix deposited by the infiltrating mesenchymal cells. Thus, by day 28, the collagen deposition in and around the tumour was no different from the unevolved SCID mice (Fig. 2b).

We also examined changes in gene expression patterns from the tumours extracted from non-evolved and evolved Black/6 and non-evolved and evolved SCID mice at the end of the study (day 28 of tumour growth).^17–19^ With a general linear model, we examined how much of the variance in gene expression of the tumours was explained by the host accession (SCID vs Black/6) that the tumours were grown in, the level of selection the host had experienced (non-evolved vs evolved) or the interaction of how tumours from different host accessions responded differently to host selection. Principal component 1 (PC1) explained 35% of the variation in genome-wide gene expression and was most closely associated with the interaction term (accession × level of host selection) in the model. In addition, the majority of transcriptional variance was attributed to the interaction between host accession and host level of selection (35.6%) followed by differences explained by host accession (19.4%), and difference explained by levels of host selection (14.8%). The tumours from the two mouse accessions differed significantly in expression of 615 genes, tumours in the two levels of selection differed in expression of 335 genes, while the interaction term explained a significant amount of variation in 1215 genes. These results support the hypothesis that the different mouse accessions elicited different responses in the tumours, and that the tumour’s response to selection for resistance in the host also depended on the host accession (Supplementary Fig. S8a, b).

**Fig. 5:**
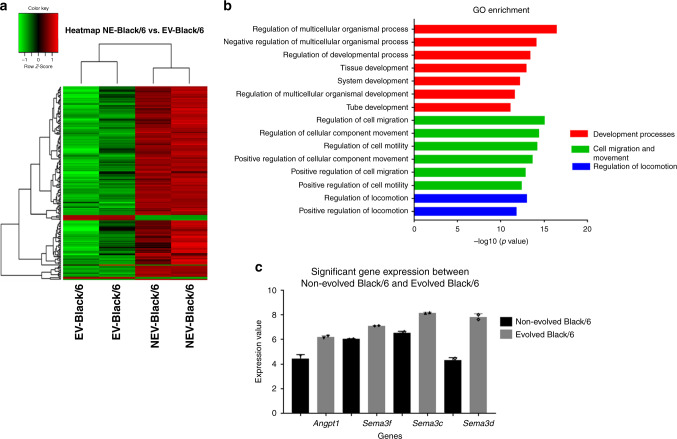
Counterstrategy of tumour cells in evolved Black/6 mice at 28 days. **a** Heat map analysis of tumour isolated from non-evolved and evolved Black/6 mice at 28 days. Two replicates of each are shown (green = downregulated genes and red = upregulated genes); normalised expression value is indicated at the top of the figure. **b** Gene ontology enrichment analysis of microarray data between non-evolved and evolved Black/6 (Top 15 processes), processes that have the same function were grouped together and colour coded. **c** The expression level of genes related to angiogenesis angiopoietin 1 (Angpt1) and semaphorin-3 (Sema3 f, c and d subtypes) (*n* = 2). NEV (non-evolved), EV (evolved).

We further explored genes that were differentially expressed between tumours grown in non-evolved Black/6 and evolved Black/6 mice (Fig. 5a, and Supplementary Fig. S9a). When classified by function, the differentially expressed genes were enriched for developmental processes, cell migration and movement, as well as cell membrane composition (Fig. 5b). The gene with the highest fold expression increase was Semaphorin 3D (Sema3D), which is implicated in the development and formation of blood vessels during angiogenesis, and the regulation of the epithelial-to-mesenchymal transition, EMT^20–22^ (Fig. 5c).

**Fig. 6:**
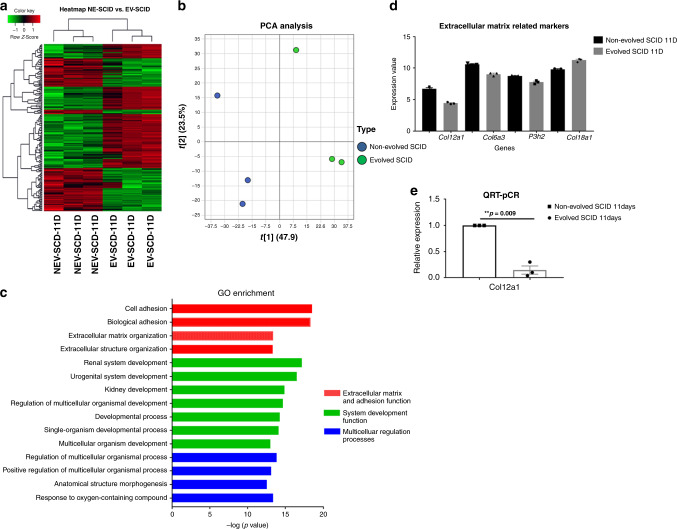
Counterstrategy of tumour cells at 11 days post injection into SCID mice. **a** Heat map and **b** principal component analysis (PCA) of genes differentially expressed between non-evolved and evolved SCID mice at early (11 days) time point. **c** Gene ontology enrichment analysis of microarray data between evolved and non-evolved SCID at 11 days (Top 15 processes). **d** Expression level of genes related to extracellular matrix pathways in evolved SCID mice compared to non-evolved SCID mice (*n* = 3). Genes are collagen subtypes (12a, 6a3 and 18a1) and poly-3-hydroxylase (P3h2). Details about regulated genes are available in the online supplements as well as raw data files. **e** QRT-PCR showing significantly (*p* = 0.009) decreased expression of Col12a1 in Evolved SCID mice (*n* = 3). Mean ± SEM is plotted. A 2-tailed unpaired Student’s *t* test was employed. NEV (non-evolved), EV (evolved).

In the SCID mice, the greatest difference in tumour size between non-evolved and evolved mice occurred at day 11. From day 11 to day 14, we saw accelerated tumour growth in the evolved scid mice, and after day 14, the tumour sizes of non-evolved and evolved mice did not differ significantly, indicating that the LL/2-Luc-M38 cells had counteracted the enhanced antitumour strategies of the Evolved SCID mouse. The LL/2-Luc-M38 cells in the evolved SCID mice appeared to eventually disrupt the increased density of the extracellular matrix. We identified gene expression differences in cancer cells harvested at day 11 (Fig. 6a, b). Most genes with increased expression in the evolved SCID mice were related to integrin binding, cell adhesion molecular binding and extracellular matrix (ECM) binding (Fig. 6c). Expression levels of extracellular matrix markers, such as collagen type VI (Col18a1), alpha, prolyl-3-hydroxylase 2 (p3h2) and collagen type XII alpha (Col12a1), were decreased in the cells isolated from evolved mice compared to non-evolved (Fig. 6d). Validation of Col12a1RT-PCR expression levels demonstrated a significant decrease (**p = 0.009) in Col12a1 as expected (Fig. 6e). Other genes were not validated since it is beyond the scope of this paper.

Tail vein injections of Lewis lung cell line in both Non-evolved and evolved SCID mice, demonstrated no development of resistance to metastasis, indicating that the resistance was specific to the subcutaneous model (Supplementary Fig. S10a–d).

## Discussion

Here we selectively bred laboratory mice to promote adaptations rendering them relatively resistant to a cancer cell population. One of our goals was to determine feasibility—could we evolve, by selective breeding, resistance in laboratory animals within a reasonably short period of time and using a relatively small number of animals? Within 10 generations, two mouse accessions (the immune-competent Black/6 and the immunodeficient SCID) evolved tumour-suppressor adaptations to Lewis lung cancer cell tumours. Such experiments could be repeated using different hosts and different tumour types to demonstrate the range of tumour-resistance strategies available in multicellular organisms. With the mice evolving resistance, we anticipated that the cancer cells would deploy observable counteradaptations in response to the hosts’ cancer-suppression mechanisms. This evolutionary tumour-host “arms race” in which hosts evolve over generations and cancer cells evolve within hosts has been anticipated theoretically,^23^ but not experimentally investigated systematically.^23^

The early suppression of tumour growth in the evolved Black/6 mice depended on changes to the innate immune system, more so than we anticipated. Furthermore, contrary to expectation, molecular studies of tumour cells’ countermeasures did not show significant changes in the expression of immune-related genes. Rather, we found decreased expression of genes associated with angiogenesis, and ATP production (and the proliferation rate), as well as increased expression of genes regulating EMT. Note that these cancer cell countermeasures did not necessarily require novel mutations (although we did not evaluate possible sequence differences). Rather, the tumour *population* deployed adaptive strategies involving changes to the intrinsic properties of individual cells, and a more global change in the vascular ecology of their tumour microenvironment.^24,25^ Collectively, these changes would impede the target immune response of the mouse to access the cancer cells.

Increased fitness is a common adaptation of prey following the introduction of a predator—simply proliferating faster than the predator can kill them.^26,27^ Hence, the LL/2-Luc-M38 cells may have increased fitness as a rapid response to the heightened predatory activity of the immune response in the evolved Black/6 mice. Reduced angiogenic signalling in the LL/2 cells grown in evolved Black/6 mice by day 11 compared to tumours in non-evolved mice (*p* = 0.0148) was likely a “niche construction” strategy. Although quantification of tumour-infiltrating immune cells was not included in our analysis, reduced blood flow may represent an immune evasion strategy by tumour cells. Reduced vasculature provides cancer cells with safety through decreased immune-cell delivery and reduced efficiency of immune-cell attack due to associated hypoxic and acidic environmental conditions.^28^

In contrast, the immune-deficient SCID mice, reduced tumour growth by increasing mesenchymal cell proliferation. This host strategy may restrict tumour growth via barriers and space limitations, and suppress cancer cell proliferation as the rapidly growing mesenchymal population competes for space and nutrients. The molecular countermeasures in the LL/2-Luc-M38 cells to the host defence manifested as increased expression of genes that produce remodelling of the extracellular matrix. This suggests that the host defence was primarily one of biomechanical restriction, and the cancer cells upregulated the means to degrade the barriers (Supplementary Fig. S11). While mesenchymal infiltration and fibrosis is commonly observed in desmoplastic clinical cancers, the role of biomechanical tumour suppression is relatively underappreciated compared to that of the immune system.

In conclusion, a growing tumour represents one of many possible outcomes from the complex eco-evolutionary interactions between cancer cells and their host organism. The role of the host in tumour suppression is well-recognised. Studies have addressed the unexpectedly low cancer rates in large (e.g. whales and elephants) or long-lived (e.g. naked mole rats) animals found in nature.^29^ Laboratory mice entirely resistant to injected cancer cells have been serendipitously discovered.^30^ Here, we investigated laboratory mice that, depending on breeding protocols, may have some evolutionary pressure for tumour suppression if their reproductive lifespan exceeds that of their ancestors living in a natural environment. We artificially imposed an increased selection force for tumour suppression by inoculating them with a rapidly growing, lethal cancer cell line, and then bred only the mice with the slowest tumour growth at 11 days after inoculation. Over 10 generations, both immunocompetent and immunosuppressed mice evolved mechanisms to suppress tumour growth during 11 days following inoculation. However, even after 10 generations, when the mice were maintained beyond the 11-day selection period, tumour growth rapidly accelerated. Thus, the host evolved tumour-suppression strategies, but the cancer cells eventually found adaptive strategies to overcome these suppression mechanisms, despite never previously encountering them.

Countering host responses is governed by novel evolutionary dynamics of cancer cells. Since cancer populations die with their host, each new tumour must re-evolve solutions to the inherited defences of the host. This would seem to be a significant disadvantage for the cancer cells. However, the cancer cells possess the same genome as the host. Thus, every antitumour strategy in the host will likely have an antidote encoded in the genome to prevent damage to normal cells. In our study, the tumour cells were from the same species though not the same accession as the host, but nevertheless, the tumours could rapidly “find” solutions to the host response within their genome.

Finally, we demonstrate that rapid phenotypic changes in cancer populations can result from changing gene expression, thus accessing information already encoded in the genome of the host species. Our results suggest this ability to acclimate, as well as evolution plays a critical role in the ability of a cancer cell to adapt to environmental conditions to which it has not been previously exposed, including treatment. The epigenetic dynamics underlying this ability to locate and then translate inactive genes will require additional investigation.

## Supplementary information

Supplemental Material

## Data Availability

All data and materials are available in this study.
