# Supplementary material for: Artificial selection for host resistance to tumour growth and subsequent cancer cell adaptations: an evolutionary arms race
Source: Br J Cancer. 2020 Oct 7;124(2):455–65. doi: 10.1038/s41416-020-01110-1 (PMC7852689; doi:10.1038/s41416-020-01110-1)
Supplement: Supplementary file 1 — Supplemental Material [file 41416_2020_1110_MOESM1_ESM.docx]

**Supplemental File**


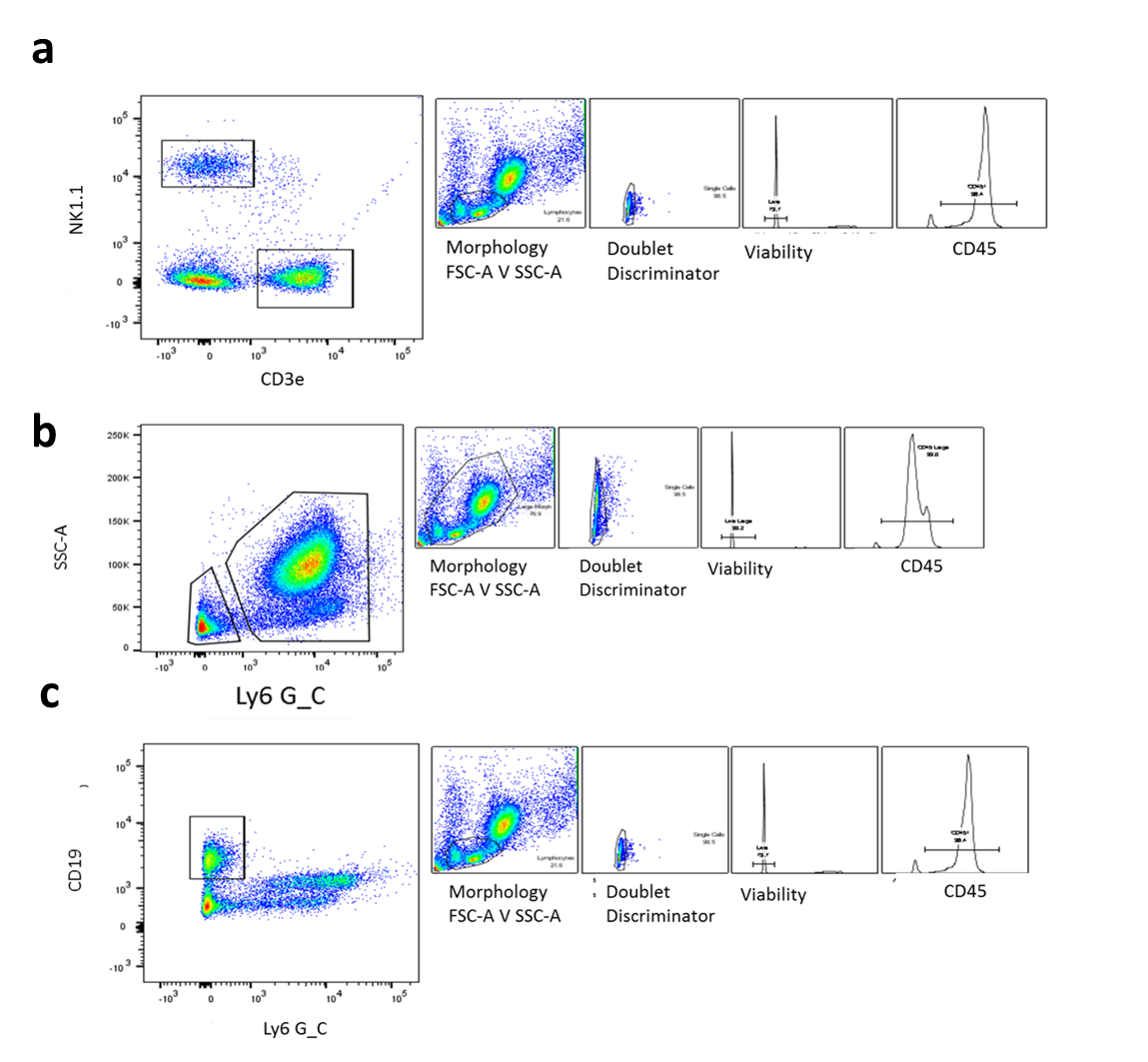


**Supplementary Fig. S1. Gating strategy for immune-phenotyping of circulating blood.** Sample gating strategy demonstrates how cells were classified for flow cytometry analysis. Morphology gating (FSC-A vs. SSC-A) was used to eliminate debris and gate on cells of interest. This was followed be elimination of doublets and dead cells as well as any CD45 negative cells for all samples. **a** T cells were determined as CD3e positive and NK1.1 negative. NK cells were determined as NK1.1 positive and CD3e negative. **b** Ly6G/C vs. SSC-A was used to enrich for cells of myeloid origin. **c** B cells were determined as CD19 positive and LY6G/C negative.


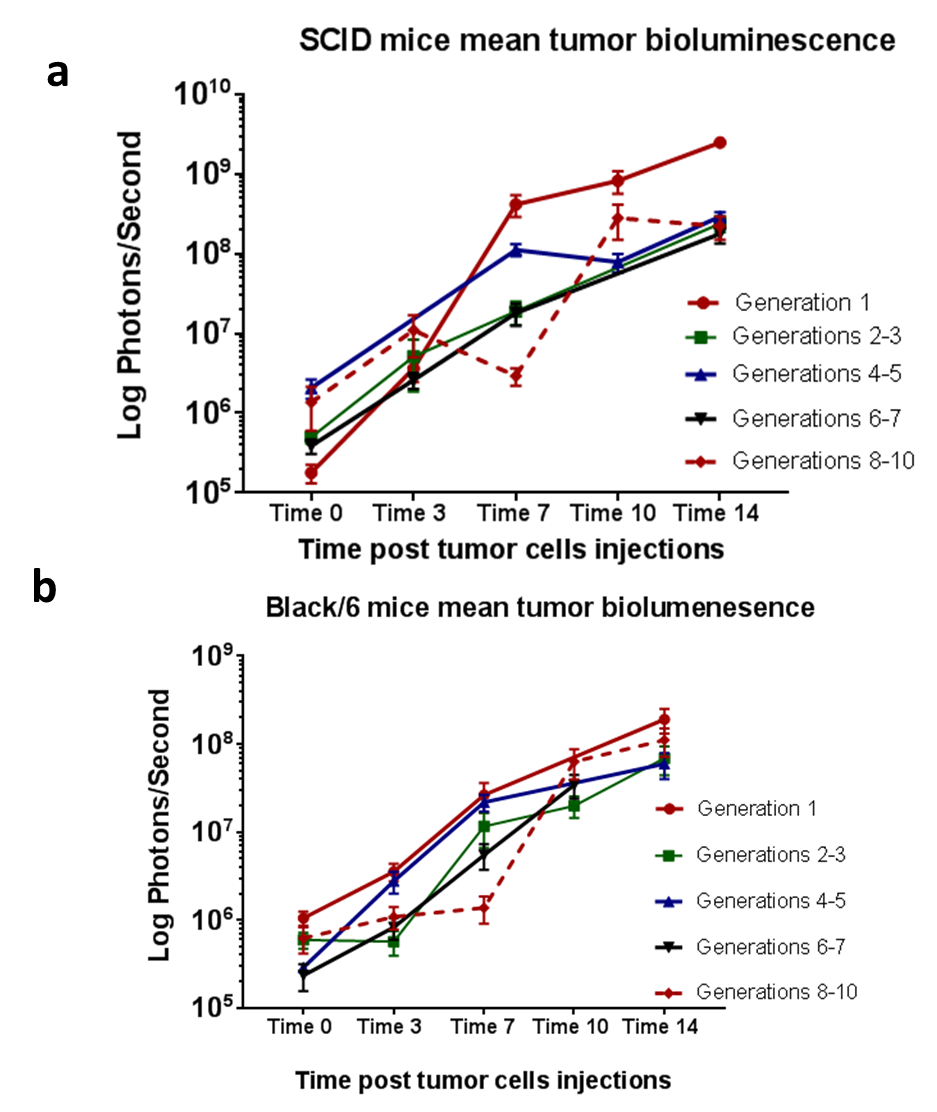


**Supplementary Fig. S2.** Quantification of tumor bioluminescent over time for generations 1 to 10 in: **a** SCID mice and **b** inBlack/6. Both accessions demonstrate a significant decrease starting at 7 days post-inoculation and at 4th generation (p<0.001 in both cases). Mean+/- SEM (n=10) is plotted with significance based on ANCOVA (analysis of covariance). Note log scale.


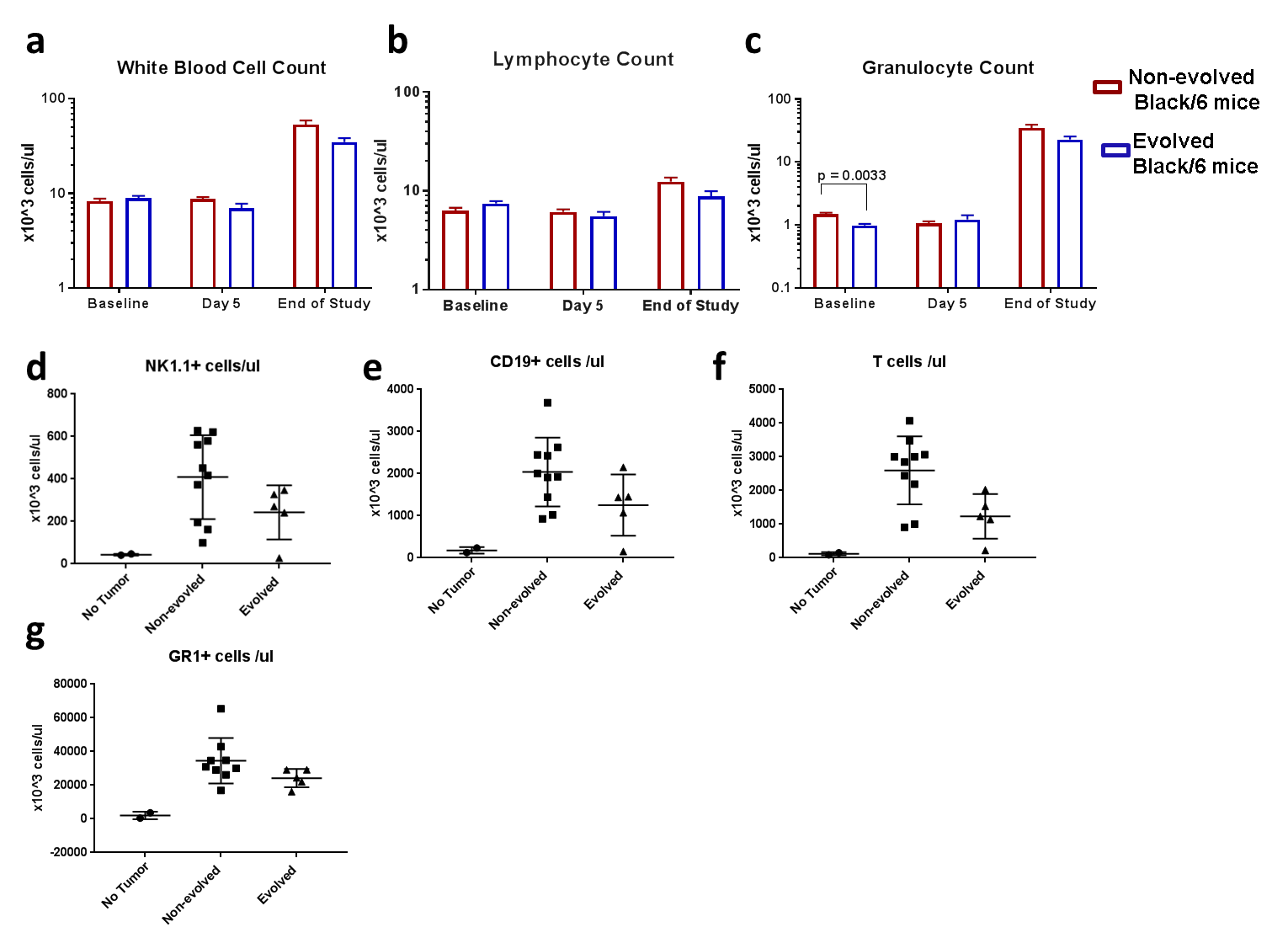


**Supplementary Fig. S3. Immune cells counts in blood**: Quantification of **a** White blood cell count, **b** Lymphocytes counts**,** and **c** granulocytes counts between Evolved mice and Non-evolved Black/6 mice, no differences are observed in white blood and lymphocytes counts, however, significant decrease in baseline in Evolved compared to Non-evolved( p=0.003) is observed. Frequency of **d** NK cells (CD3**^-^**,NK1.1**^+^**) **e** B cells (CD3**^-^**, NK1.1**^-^** CD19**^+^**) **f** T cells (NK1.1**^-^**, CD3e**^+^**) and **g** Gr1**^+^** cells performed by flow cytometry demonstrated no change in NK1.1+, CD19+ or Gr1+ cells between the Non-evolved and Evolved Black/6 mice. T cells were slightly decreased (p=0.0172) in the Evolved mice after 25 days of tumor growth.


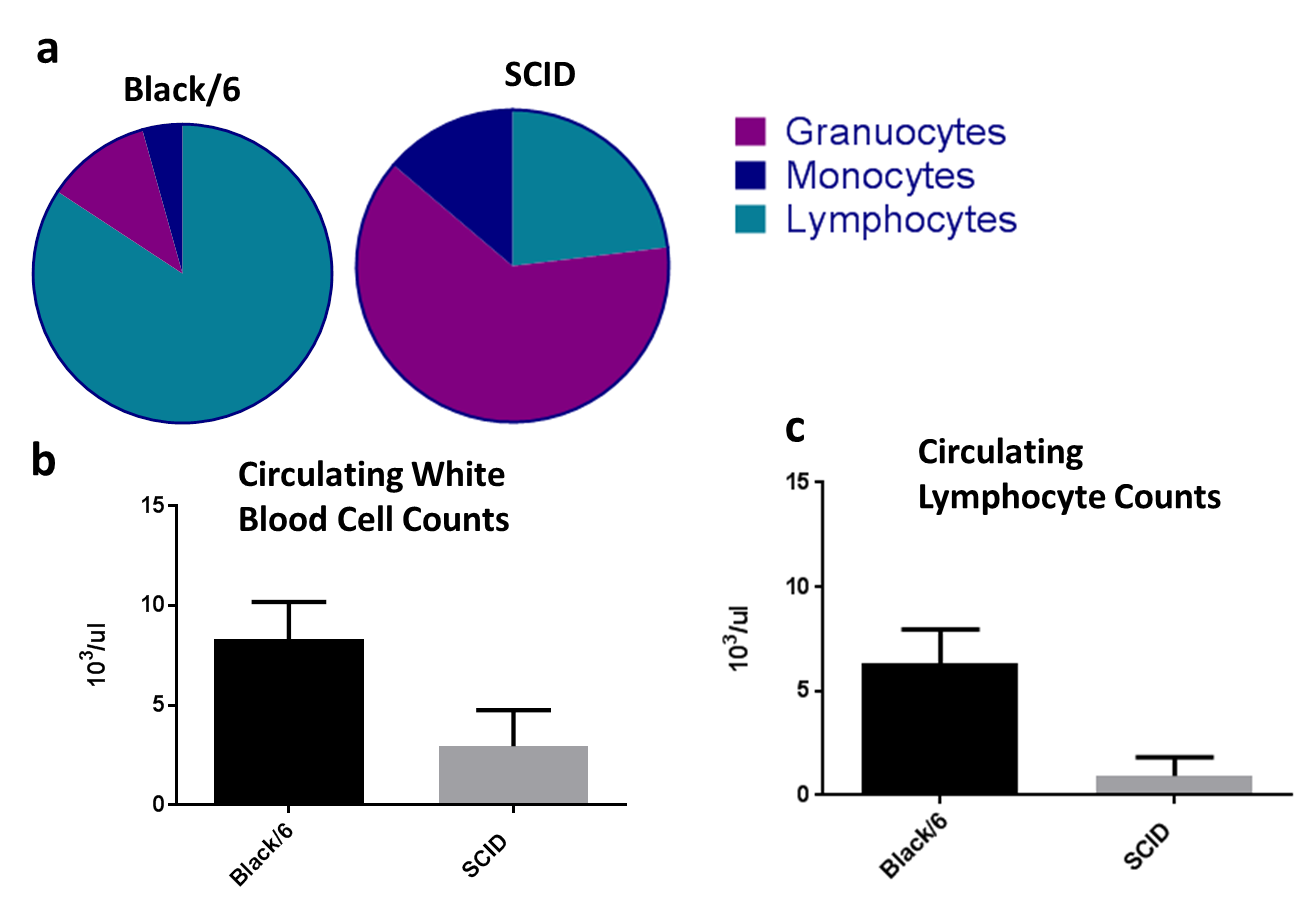


**Supplementary Fig. S4. Immune cell frequencies in Non-evolved Black/6 and SCID mice**: **a** Black mice have an intact lymphocyte compartment. **b** SCID mice have decreased white blood cell counts and **c** lymphocyte counts compared to Black/6 mice (p<0.0001 and p<0.001 respectively).


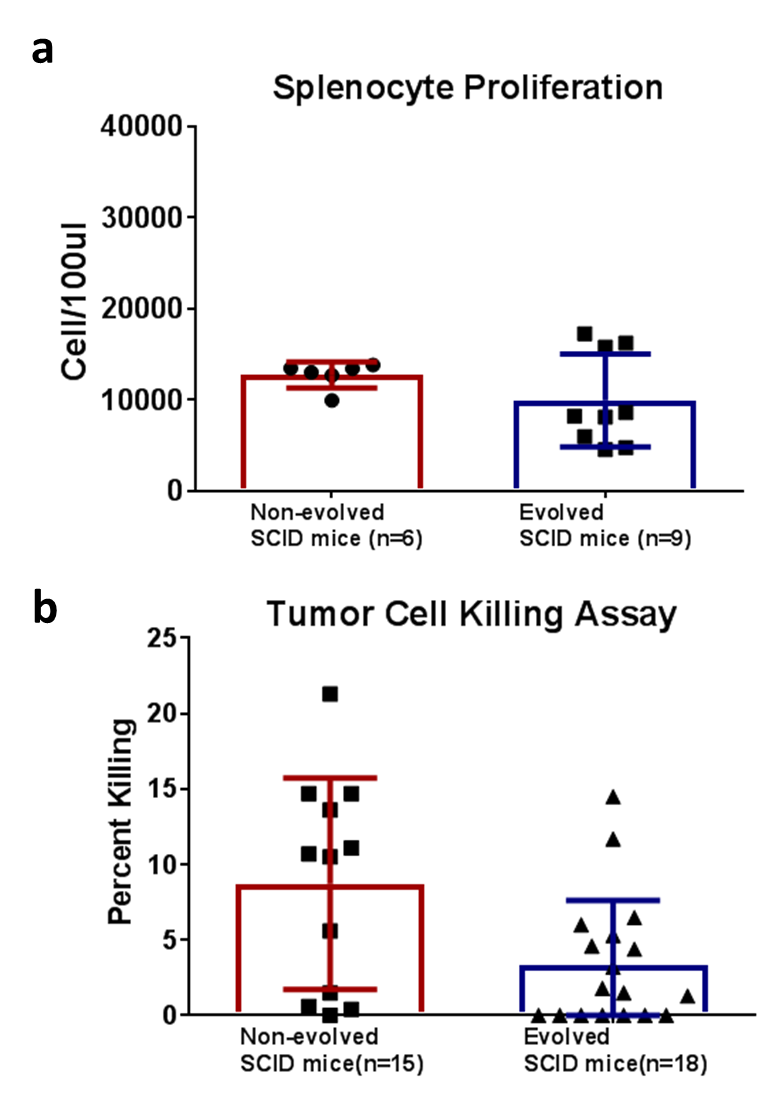


**Supplementary Fig. S5. a** Ex-vivo quantification of surviving splenocytes, and **b** tumor cell killing assays demonstrated no differences between Non-evolved and Evolved SCID mice. Mean+/- SEM is plotted with significance based on two-tailed unpaired *t* -test was employed.


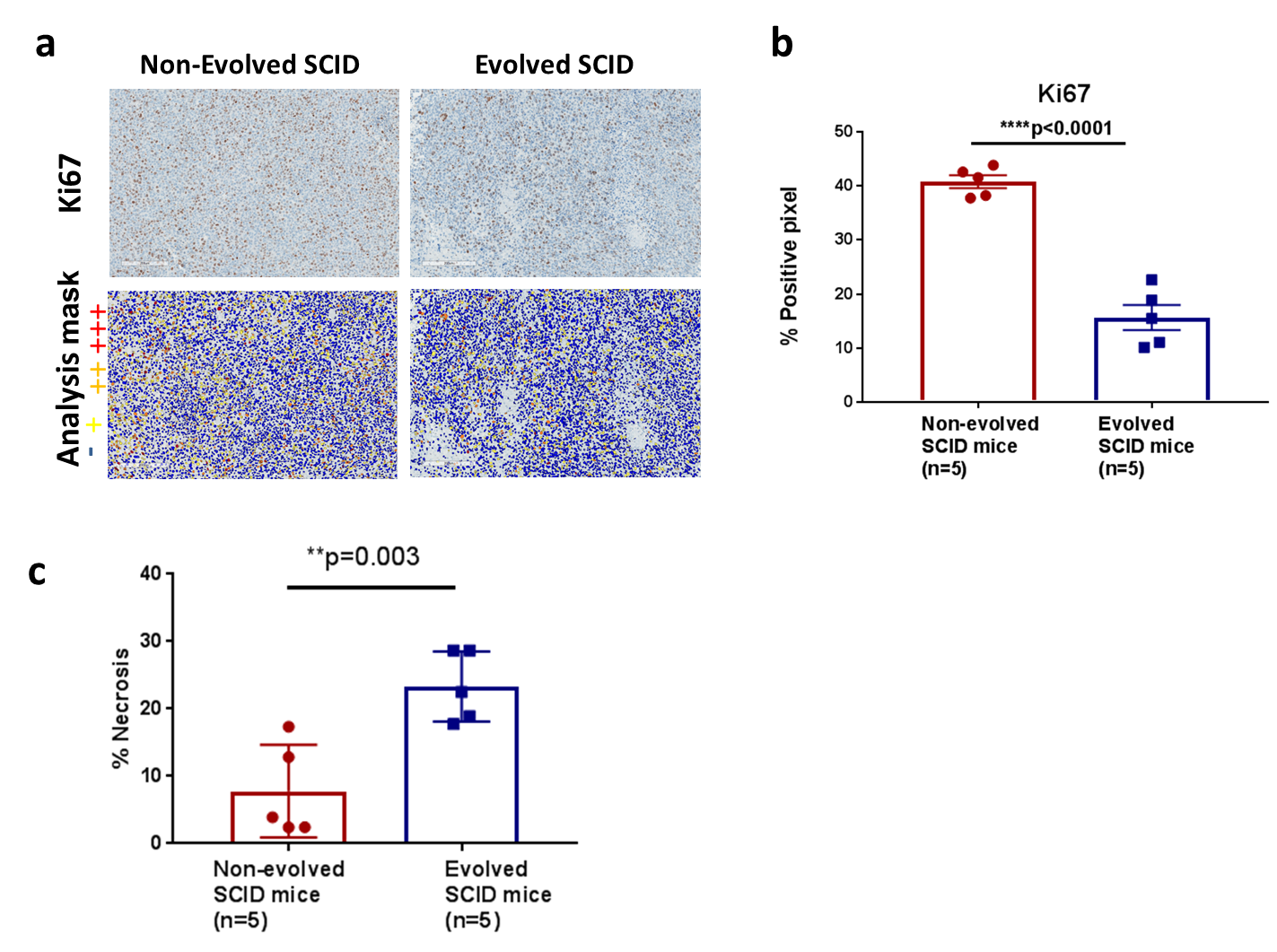


**Supplementary Fig. S6. Tumor cell proliferation and necrosis at day 11, a** Immunohistochemistry staining and **b** quantification of Ki67 in tumors in Evolved SCID mice. Representative images of the tumor (upper panel) as well as a positive mask (lower panel). Percent Ki67 positive pixels were quantified over the entire viable area of tumor cross-section. Mean+/- SEM is plotted. Scale bar in IHC images is 200µm**. c** Percent necrosis of tumor cells in Non-evolved SCID mice and Evolved SCID mice. Mean+/- SEM is plotted.


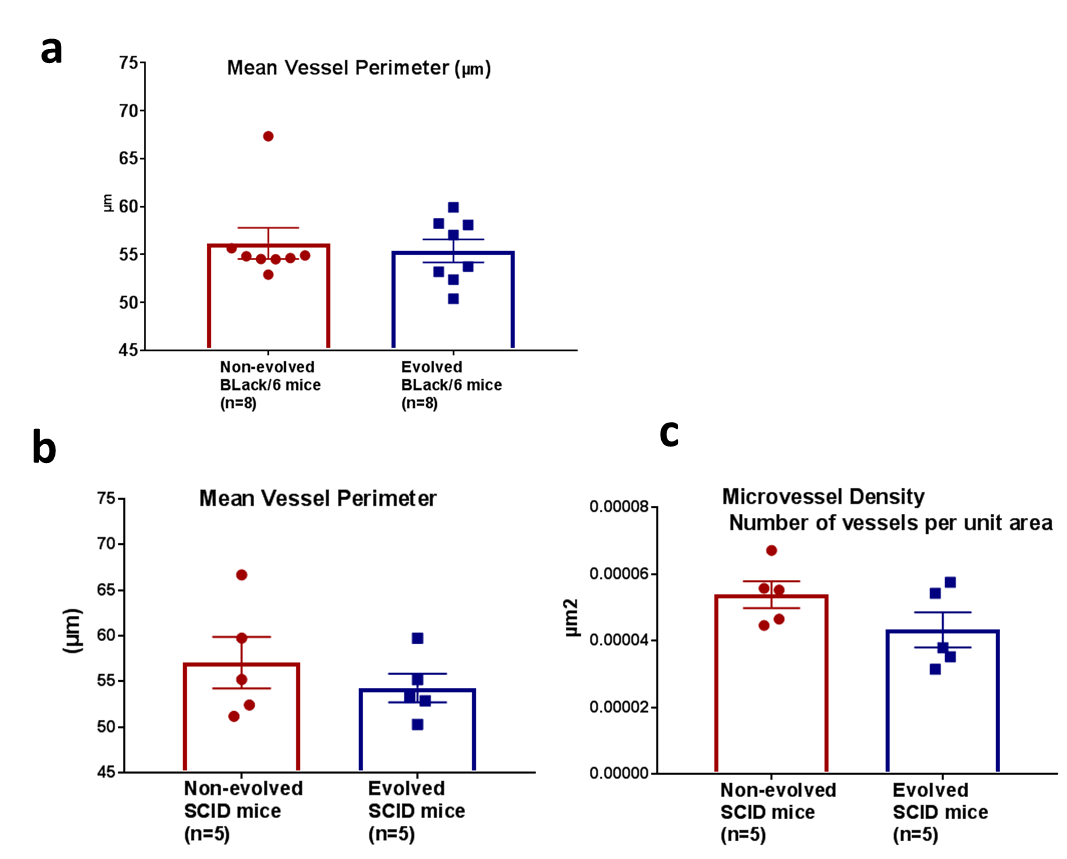


**Supplementary Fig. S7. Angiogenesis: Microvessels in tumors at day 11**. **a** Quantification of mean vessel perimeter in tumors of Non-evolved Black/6 mice and in Evolved Black/6 mice. There was no significant difference. **b, c** Quantification of microvessel density (MVD), and mean vessel perimeter for Non-evolved SCID mice and Evolved SCID mice. There was no significant difference. Mean +/- SEM are shown with significance based on two-tailed t-test.


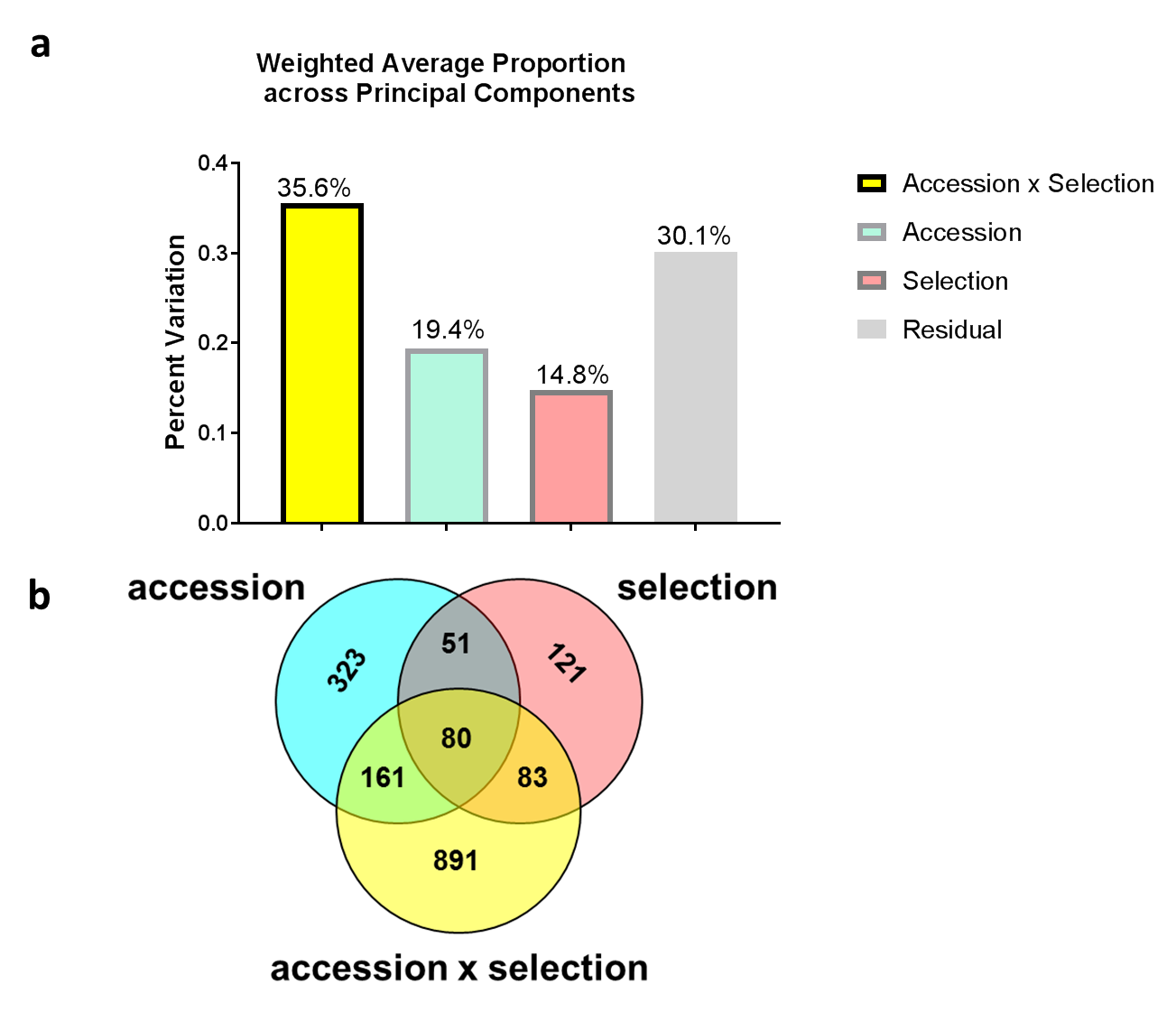


**Supplementary Fig. S8. Components of transcriptional variance in tumor cells, a** percent of the transcriptional variance in tumors explained by which host accession (SCID vs Black/6) was infected, the level of host selection (Non-evolved vs Evolved) and the interaction of tumors from the two host accessions and the level of host selection (Accession x Selection). **b** number of genes that significantly differ between tumors from Black/6 and SCID accessions, tumors from Evolved vs Non-evolved mice (selection status), and that show an accession-by-selection status interaction.


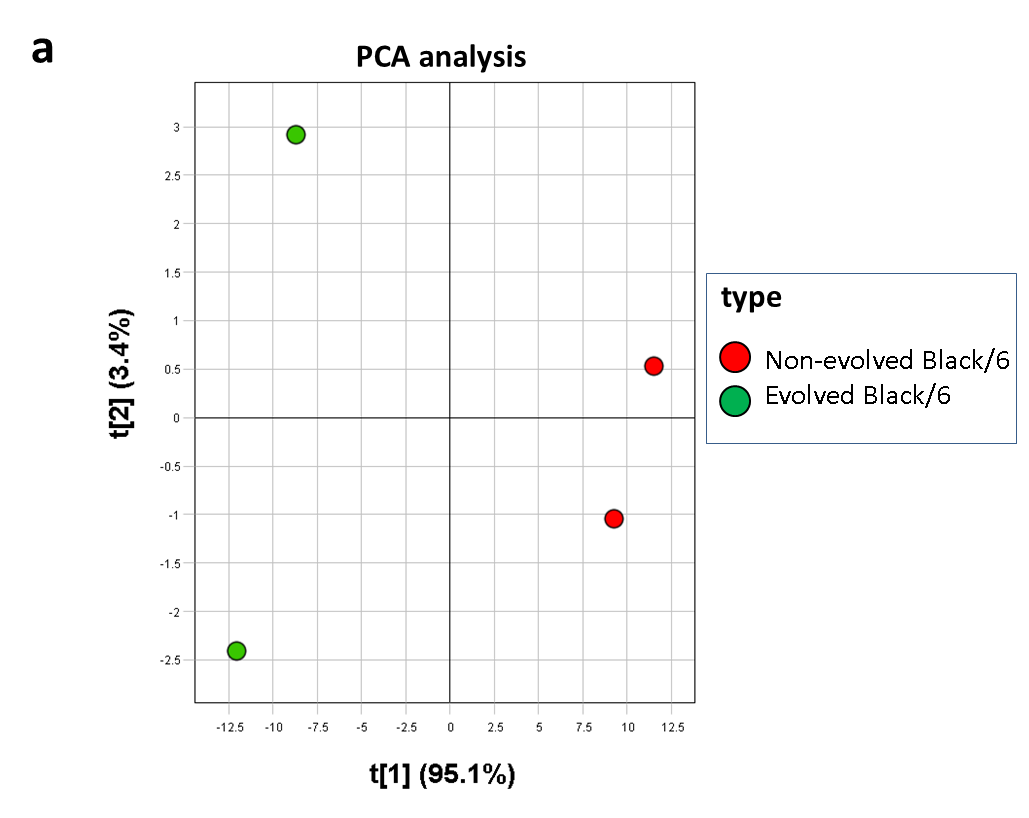


**Supplementary Fig. S9. Counterstrategy of tumor cells in Evolved Black/6 mice at 28 days: Gene Expression. a** Principal Component Analysis (PCA) of tumor isolated from Non-evolved and Evolved SCID mice at 28 days


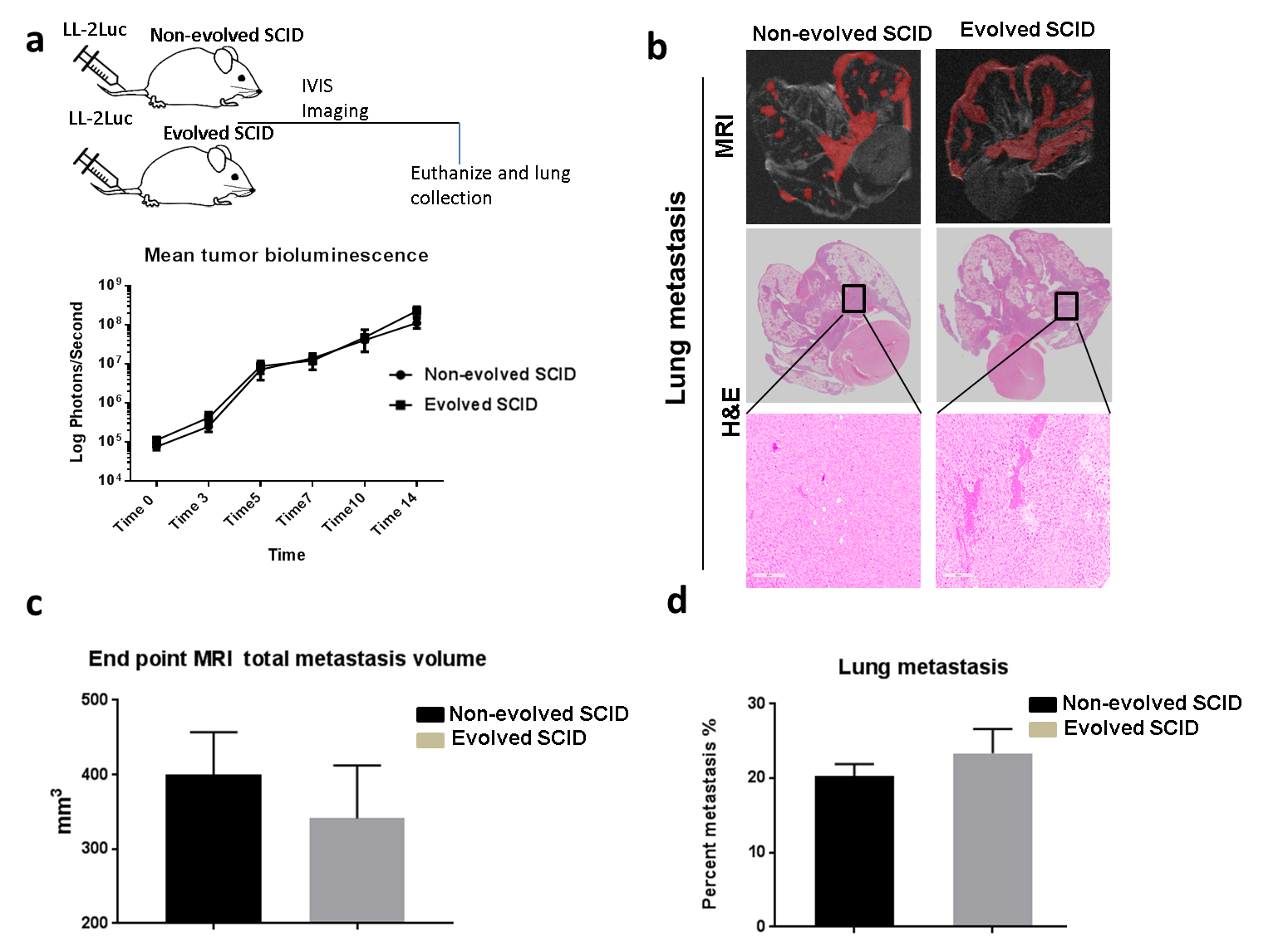


**Supplementary Fig. S10. No experimental metastasis. - a**. Mean tumor bioluminescence in Non-evolved and Evolved SCID mice (n=5) after induction of experimental metastases, indicating no difference in metastases in the Non-evolved -SCID cohort than in the Evolved SCID mice (note log scale).**b** MRI (Top) and H&E images(middle and lower) Images of the lung for one representative mouse from each group. **c** Total lung metastasis volume measured by MRI, and **d** Percent lung metastasis quantification of H&E. Mean+/- SEM is plotted. A two-tailed unpaired Student’s *t*-test was employed.


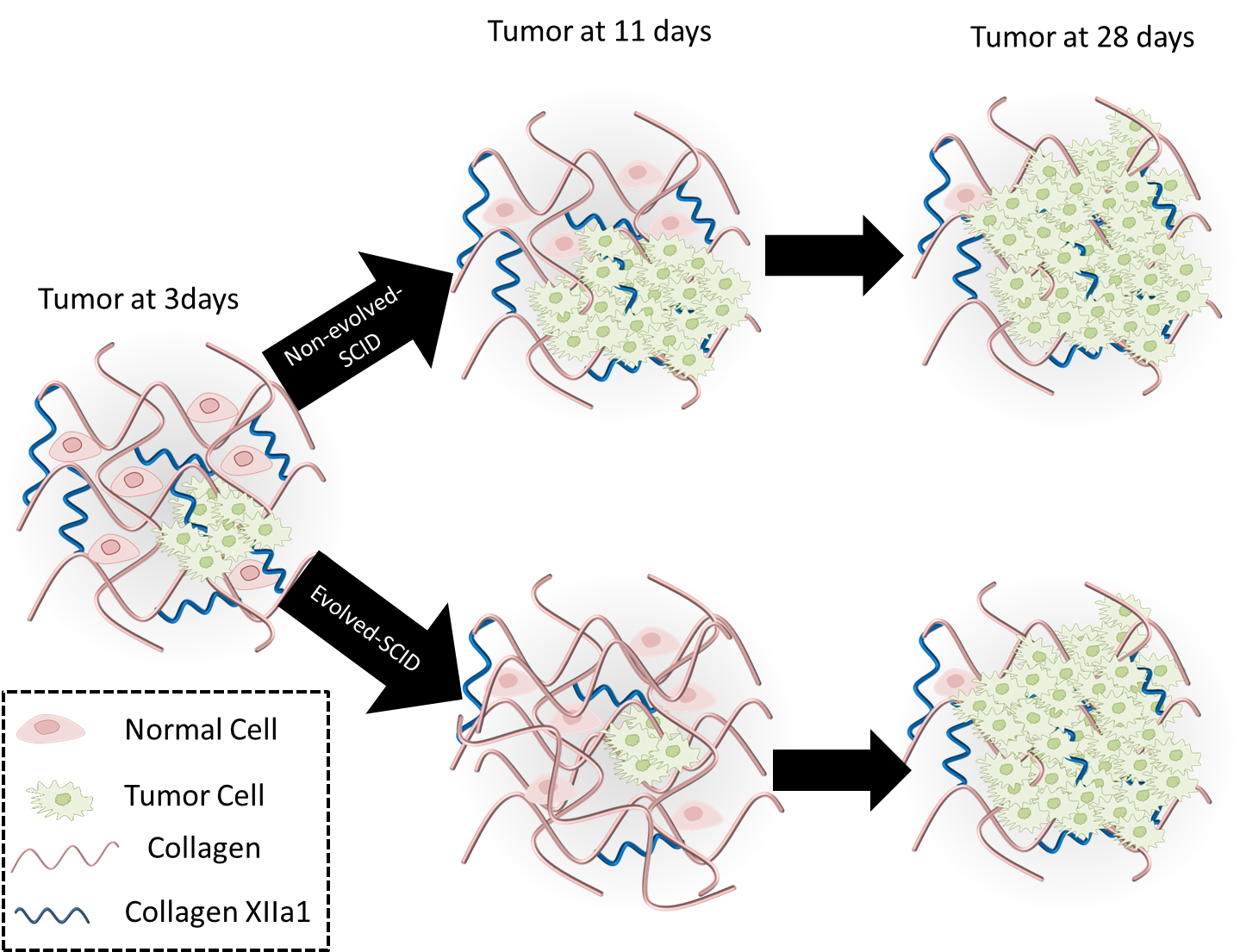


**Supplementary Fig.S11.** **Model of tumor growth in Evolved SCID mice versus Non-evolved SCID mice**, showing the acclimation of the tumor cells to increased collagen at early time point of evolved mice by decreasing the integrin binding collagen XIIa1.
